# Prevalence and risk of thyroid disease among adult primary aldosteronism patients: a systematic review, meta-analysis, and trial sequential analysis

**DOI:** 10.3389/fendo.2025.1614789

**Published:** 2025-10-17

**Authors:** Wansong Hu, Yingxing Wu, Ping Li

**Affiliations:** Department of Cardiovascular Medicine, The Second Affiliated Hospital, Jiangxi Medical College, Nanchang University, Nanchang, China

**Keywords:** primary aldosteronism, thyroid, prevalence, meta-analysis, risk

## Abstract

**Background:**

Primary aldosteronism (PA), the most prevalent curable secondary hypertension, and thyroid diseases (the second most common endocrine disorder) are increasingly linked, yet their mechanistic connections remain unclear.

**Design and methods:**

Four databases (PubMed, Embase, Web of Science, Cochrane Library) were searched for case-control studies. Random-effects meta-analysis and subgroup analyses for thyroid disease subtypes were performed. Sensitivity/trial sequential analyses and Begg’s test evaluated robustness/publication bias.

**Results:**

Our meta-analysis included five case-control studies, encompassing 1,368 patients with primary aldosteronism (PA) and 6,774 controls. While the overall prevalence of thyroid diseases was higher in PA patients (OR: 1.33, 95% CI: 1.03-1.71, p=0.03), subgroup analysis revealed that this association was primarily driven by a significantly increased prevalence of thyroid nodules (OR: 1.85, 95% CI: 1.23-2.80, p=0.003). No statistically significant associations were found between PA and other specific thyroid conditions, including hyperthyroidism, hypothyroidism, thyroiditis, or thyroid cancer (all p > 0.05).

**Conclusions:**

This first meta-analysis demonstrates a significant PA-thyroid disease association. The elevated overall risk of thyroid disease in PA patients appears to be largely attributable to the high burden of thyroid nodules. These findings suggest that patients with PA may benefit from targeted screening for thyroid nodules.

## 1 Introduction

Primary aldosteronism (PA) represents the most prevalent form of endocrine hypertension and is also the leading cause of secondary hypertension (1, 2). The two main subtypes of PA are aldosterone-producing adenomas (APAs) and idiopathic hyperaldosteronism (IHAs) (3). The reported prevalence of PA exceeds 5 percent, potentially surpassing 10 percent among patients with hypertension (4, 5). Furthermore, individuals with PA exhibit a higher likelihood of developing cardiovascular and metabolic complications compared to those with essential hypertension (6, 7). Despite the clinical significance of PA, research exploring its association with other endocrine disorders remains limited, often confined to a few case reports (8).

Thyroid diseases hold a significant position within the endocrine system, encompassing conditions such as hyperthyroidism (e.g., Graves’ disease), hypothyroidism (e.g., Hashimoto’s thyroiditis), and thyroid nodules (9–12). Recently, advancements in endocrine research have prompted an increasing number of scholars to investigate the potential link between primary aldosteronism (PA) and thyroid diseases (13–15). For example, studies suggest that hyperthyroidism may be associated with elevated aldosterone levels, potentially due to the direct or indirect effects of thyroid hormones on adrenal cortical function (16–18). Additionally, there exists an interaction between thyroid hormones and the renin-angiotensin-aldosterone system (RAAS), which may further influence the pathogenesis of PA (19–21).

Although current research provides preliminary evidence for a relationship between primary aldosteronism (PA) and thyroid disorders, most studies are cross-sectional, lacking long-term follow-up data and involving limited sample sizes. These limitations have led to ongoing debates regarding the results. Therefore, our systematic review and meta-analysis aimed to synthesize the findings from studies in this area and assess the prevalence and potential associations between primary aldosteronism and thyroid disorders.

## 2 Methods

### 2.1 Protocol and registration

This systematic review and meta-analysis have been reported with reference to the Preferred Reporting Items for Systematic Reviews and Meta-Analyses (PRISMA) guideline and has been prospectively registered in PROSPERO (CRD42025629905) (22).

### 2.2 Search strategy and selection criteria

Four databases (PubMed, Embase, Cochrane Library, and Web of Science) were searched for this systematic literature review and meta-analysis from the inception of the databases through December 1, 2024. The search terms included those related to primary aldosteronism (e.g., hyperaldosteronism, aldosteronism, Conn syndrome) and thyroid diseases (e.g., thyroid function, hypothyroidism, hyperthyroidism, and thyroid cancer). The detailed search strategy is provided in the **Supplementary Material**. Literature meeting the inclusion criteria were exported to EndNote Desktop version X9, and duplicates were automatically removed. The studies were then screened by title and abstract, followed by full-text review according to the eligibility criteria. Two authors (WH, YW) independently participated in all stages of screening. Disagreements in the screening process were resolved through consensus discussions.

### 2.3 Eligibility criteria

All studies published in English were included as long as they met the following basic eligibility criteria.

Basic eligibility criteria were met:

1. The study was conducted in patients older than 18 years of age with a confirmed diagnosis of primary aldosteronism.
2. The prevalence of thyroid disease in patients with primary aldosteronism was compared with the prevalence in the control group.
3. PA were tested for thyroid diseases prior to the administration of therapy.

The following studies were excluded:

1. Cases of primary aldosteronism were defined on the basis of clinical suspicion rather than a definitive diagnosis.
2. Tested for thyroid disease after treatment.
3. Did not compare the prevalence of thyroid disease between PA and non-PA controls.
4. Case reports, editorials, reviews, and articles in non-English languages were excluded.

### 2.4 Quality assessment

Two authors (WH and YW) completed a quality assessment of all included studies using the Newcastle-Ottawa Scale (NOS). The NOS assesses the quality of cohort and case-control studies according to three categories. They are (a) “Selection”, which includes four assessment items with a maximum score of four stars; (b) “Comparability”, which includes one assessment item with a maximum score of two stars; and finally, (c) “Outcome “, consisting of three assessment items with a maximum score of three stars (23). The disagreements were resolved in a consensus meeting.

### 2.5 Data extraction

Two reviewers, WH and YW, extracted data in a pre-designed Excel sheet that included the items: authors, year, country of study, mean age, gender distribution, and data used to calculate prevalence estimates such as the total number of people with PA in the sample and the number of people with thyroid diseases. In the meta-analysis, the prevalence of different thyroid disorders among PA patients and controls was also extracted.

### 2.6 Statistical analysis

The primary endpoint of this study was the prevalence of thyroid disease in patients with primary aldosteronism. Combined prevalence estimates and 95% confidence intervals (CIs) were calculated using a random-effects model with logit transformations. Random-effects models account for study heterogeneity and provide more conservative estimates of overall effect sizes. For all models, the Cochrane’s Q statistic and *I^2^* were calculated to assess heterogeneity. Heterogeneity was categorized as low (<40%), moderate (40-60%), high (70-90%), or substantial (>90%) according to *I²*values. Each thyroid disorder was analyzed in a separate subgroup, and pooled odds ratios (ORs) were calculated for each subgroup as well as for all subgroups combined. To assess the impact of individual studies on the overall effect, sensitivity analyses were performed by omitting one study at a time. For publication bias, funnel plot inspection and Egger and Begg regression tests were conducted. A p-value <0.05 was considered statistically significant. Statistical analyses were performed using Cochrane Review Manager v5.4 and Stata 18. The results of this meta-analysis were reported in accordance with the Preferred Reporting Items for Systematic Reviews and Meta-Analyses (PRISMA) guidelines.

### 2.7 Trail sequential analysis

To assess the risk of false-positive or false-negative results in the meta-analysis, we conducted trial sequential analyses (TSAs) using TSA software (version 0.9.5.10 beta; Copenhagen Trial Unit, Clinical Intervention Research Center, Rigshospitalet, Copenhagen, Denmark). The required information size (RIS) was calculated with an alpha risk of 5%, a beta risk of 20%, and a two-sided border type. When the cumulative Z-curve enters the null zone or crosses the TSA boundaries, the intervention effect is considered to be sufficiently supported by evidence. If the Z-curve does not cross any boundaries and does not reach the required information size, the evidence is insufficient to draw any conclusions.

## 3 Results

### 3.1 Study search and selection

**Figure 1:**
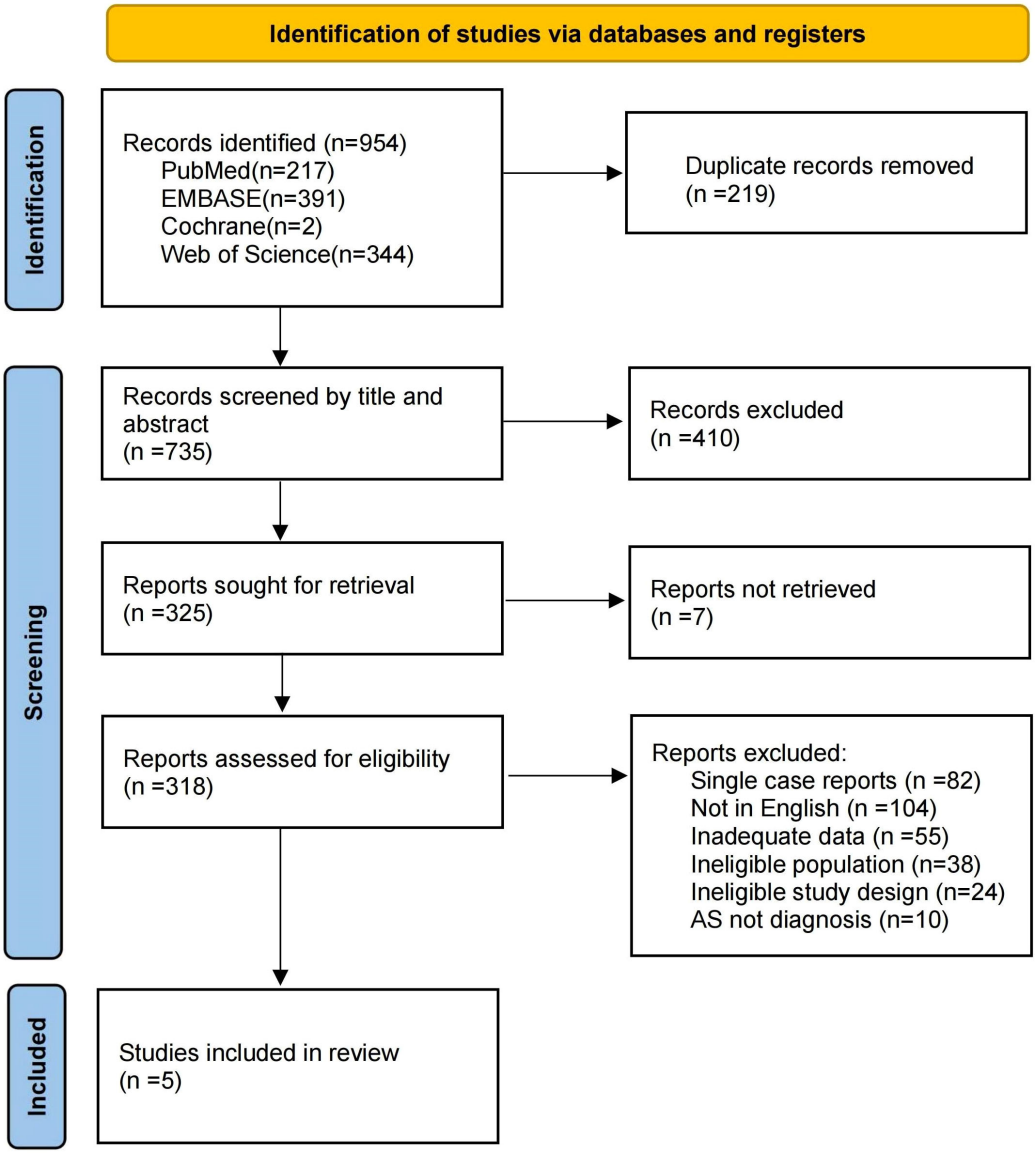
PRISMA flowchart of the study selection.

A total of 954 records were identified from database and electronic sources. After removing duplicates, 735 records were screened, and 410 studies were excluded based on title and abstract review. The remaining studies were assessed for eligibility according to predefined criteria. Ultimately, five studies were included in the meta-analysis. The study selection flowchart is shown in **Figure 1**.

### 3.2 Risk of bias assessment

All included studies were assessed for risk of bias by the NOS scale as shown in **Table 1**. The results showed that all studies had high quality and low risk of bias. Total score ranged from 7 to 9.

**Table 1:** Risk of bias across the studies; NOS scale.

Study	Selection	Representativeness of the cases	Selection of controls	Definition of control	Comparability	Exposure	Same method of ascertainment in cases and controls	Non-response rate	Total quality score
	Definition of cases				Comparability of cases and controls based on the designs or analysis	Assessment of exposure			
Sabbadin et al.,2017 (24)	1	1	1	1	2	1	0	1	8
Maiturouzi et al.,2024 (13)	1	1	1	1	0	1	1	1	7
Turchi et al.,2011 (25)	1	1	1	1	1	1	1	1	8
Armanini et al.,2003 (26)	1	1	1	1	1	1	0	1	7
Matrozova et al.,2019 (27)	1	1	1	1	2	1	1	1	9

### 3.3 Characteristics of the included studies

Five studies with a total of 1368 PA and 6774 controls were included (13, 24–27). Four studies were from Europe and one from Asia. All participants were adults, with a mean age between 50 and 55 years. In total, the studies showed that the female PA population was larger than that of women. A detailed description of the population characteristics in each study is presented in the **Table 2** below.

**Table 2:** Descriptive characteristics of the included studies.

Author and year	Study country	Sample size	Mean age	Gender distribution (Female: Male)	Prevalence of thyroid disease in PA cases	PA criteria
Sabbadin et al.,2017 (24)	Italy	Cases:80(32 with APA and 48 with IHA) Control:369	Cases:55.4 Control:51.7	Cases: 1:1 Control:1.1:1	20%	Upright serum aldosterone/upright PRA ratio (ARR) [30 (ng/dL)/[ng/(mL/h)] and the diagnosis was confirmed with the captopril test and the lack of aldosterone suppression after saline load
Maiturouzi et al.,2024 (13)	China	Cases:1023(327 with APA and 696 with IHA) Control:6138	Cases:48.2 Control:48.2	Cases:0.86:1 Control:0.85:1	74%	The positive screening test was defined as PRA < 1 ng/mL/h and PAC ≥ 12 ng/dL or ARR ≥ 20 ng/dL per ng/mL/h. PA was defined if post-SIT PAC was >10 ng/dL.
Turchi et al.,2011 (25)	Italy	Cases:92(33 with APA and 59 with IHA) Control:96	Cases:51 Control:50	Cases:0.77:1 Control:1.18:1	66%	PA was suspected for an upright serum aldosterone/upright plasma– renin activity (PRA) ratio >40 (ng/dl)/(ng/ml/h), together with an upright aldosterone value greater than 15ng/dl, and confirmed with an aldosterone value >7ng/dl after a 4-h intravenous saline infusion
Armanini et al.,2003 (26)	Italy	Cases:80(40 with APA and 40 with IHA) Control:80	Cases:53.0 Control:50.5	Cases:1.5:1 Control:1.35:1	60%	The diagnosis was made by measuring PRA, aldosterone, aldosterone/PRA ratio in the upright position, and aldosterone after a saline suppression test
Matrozova et al.,2019 (27)	Bulgaria	Cases:93(40 with APA, 51 with IHA and 2 undetermined) Control:91	Cases:53.8 Control:52.4	Cases:1.38:1 Control:2.37:1	97%	The diagnosis of PA was considered if the aldosterone was> 330 pmol/l after the oral administration of 50 mg of Captopril,while the patient has been sitting for 90 minutes

### 3.4 Meta-analysis on the odds ratio of thyroid diseases in PA

A total of five studies were included to compare the combined prevalence of thyroid disorders. Patients with primary aldosteronism had different types of thyroid disease. We divided subgroups by disease and performed stratified analyses. The results of the study are as follows:

1. Hypothyroidism: No statistically significant association was found between PA and hypothyroidism [OR: 0.95, 95% CI: 0.59–1.53, *p*-value: 0.84, *I^2^* =40%].
2. Hyperthyroidism: The analysis showed no statistically significant association between PA and hyperthyroidism [OR: 1.80, 95% CI: 0.58–5.62, *p*-value: 0.31, *I^2^* =59%].
3. Thyroiditis: No statistically significant association was found between PA and thyroiditis [OR: 1.47, 95% CI: 0.19–11.26, *p*-value: 0.71, *I^2^* =54%].
4. Thyroid cancer: There is no higher prevalence of thyroid cancer in PA as compared to controls [OR: 0.97, 95% CI: 0.40–2.33, *p*-value: 0.94, *I^2^* =0%].
5. Thyroid nodules: Significantly higher prevalence of thyroid nodules in PA as compared to controls [OR: 1.85, 95% CI: 1.23–2.80, *p*-value: 0.003, *I^2^* =68%].

### 3.5 The pooled prevalence of thyroid disease in PA

**Figure 2:**
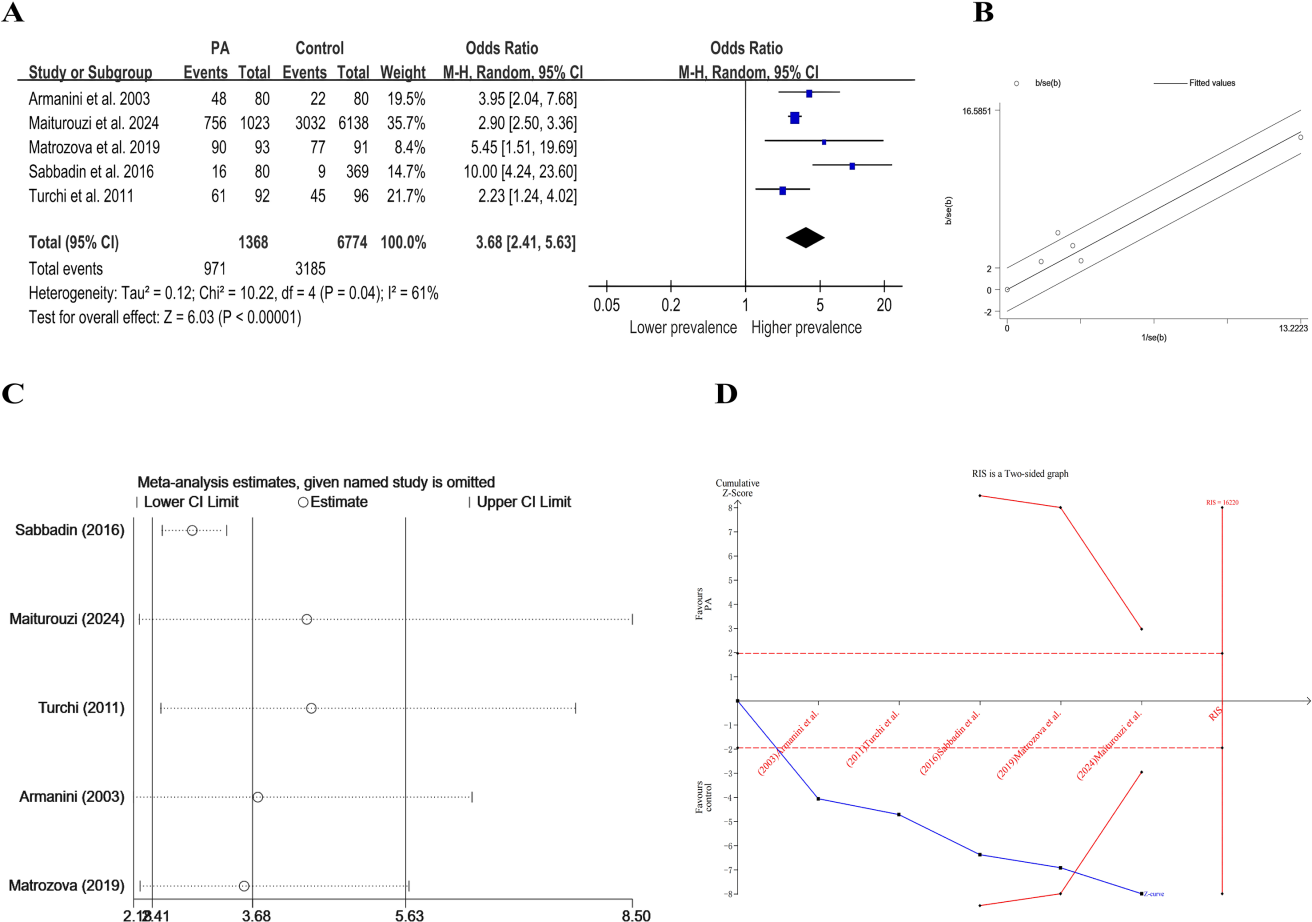
**(A)** Forest plot of event rate of overall thyroid disease among PA patients; **(B)** Galbraith plot; **(C)** Sensitivity analysis; **(D)** TSA analysis.

**Figure 3:**
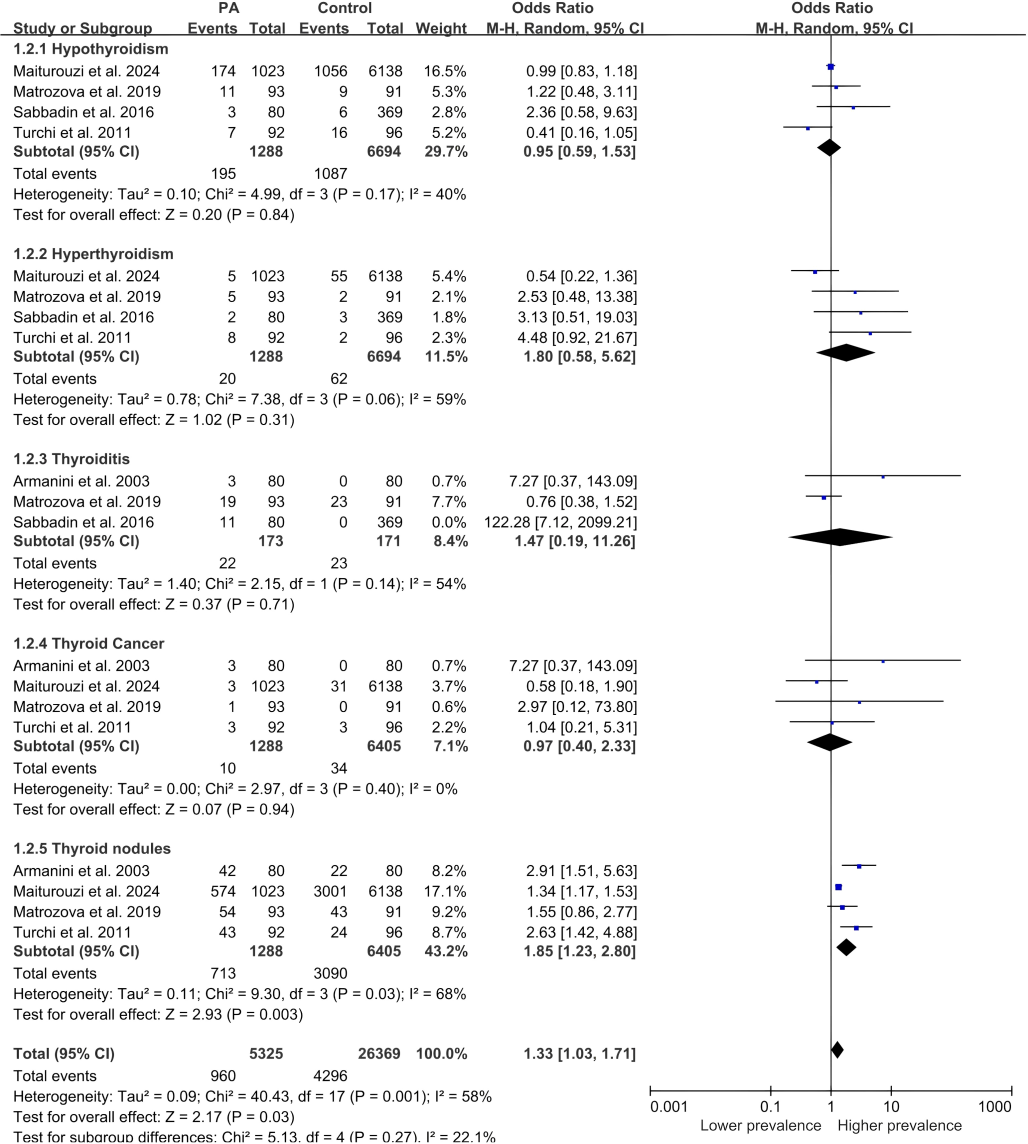
Pooled odds ratio of thyroid disease in PA and controls.

**Figure 2A** shows a forest plot of thyroid disease in patients with PA. The pooled prevalence of any thyroid disease was significantly higher in the PA group than in controls (OR: 3.68, 95% CI: 2.41-5.62, p < 0.00001). However, *I^2^* and Galbraith (**Figure 2B**) suggested some heterogeneity in findings, we therefore did further subgroup and sensitivity analyses. Subgroup analysis demonstrated that this overall difference was predominantly attributable to a significantly higher prevalence of thyroid nodules in PA patients (OR: 1.85, 95% CI: 1.23-2.80, p=0.003; **Figure 3**). In contrast, the prevalences of hyperthyroidism, hypothyroidism, thyroiditis, and thyroid cancer did not differ significantly between groups.

### 3.6 Sensitivity analysis and publication bias

In order to assess the impact of individual studies on the overall combined effect size, sensitivity analyses were performed using an exclusion-by-exclusion approach, and the detailed results are shown in **Table 3** and **Figure 2C**. Sensitivity analysis of subgroups was showed in **Supplementary Figures 1–5**. Although the pooled ORs were altered in the subgroups of hypothyroidism, hyperthyroidism, thyroiditis, and thyroid cancer, there was no significant change in the ORs in the subgroups of overall thyroid disorders and thyroid nodular disorders, a finding that suggests a consistency of results across studies. Further studies are needed to provide robust evidence. Nonetheless, there does seem to be an association between primary aldosteronism and thyroid disease. This is evidenced by the robustness of the study results.

**Table 3:** Sensitivity analysis on the effect of exclusion of each study on subgroup and pooled findings.

Group Study excluded	Hypothyroidism	Hyperthyroidism	Thyroiditis	Thyroid cancer	Thyroid nodules	Overall thyroid disease
	OR (95% CI)					
None	0.95 (0.59,1.53)	1.80 (0.58,5.62)	7.33 (0.22,245.59)	0.97 (0.40,2.33)	1.85 (1.23,2.80)	3.68 (2.41,5.63)
Sabbadin et al.	0.87 (0.54,1.41)	1.61 (0.40,6.51)	1.47 (0.19,11.26)			2.92 (2.54,3.36)
Maiturouzi et al.	0.97 (0.37,2.55)	3.33 (1.27,8.76)		1.80 (0.49,6.64)	2.23 (1.51,3.31)	4.37 (2.25,8.50)
Turchi et al.	1.01 (0.85,1.20)	1.34 (0.39,4.60)		1.36 (0.28,6.61)	1.66 (1.09,2.54)	4.43 (2.52,7.78)
Armanini et al.			8.22 (0.03,2036.46)	0.80 (0.32,2.00)	1.61 (1.11,2.35)	3.75 (2.18,6.47)
Matrozova et al.	0.90 (0.44,1.81)	1.71 (0.39,7.53)	30.93 (1.93,494.66)	0.96 (0.33,2.81)	2,03 (1.13,3.63)	3.58 (2.26,5.67)

Funnel plot analysis of thyroid disease in patients with primary aldosteronism is shown in **Supplementary Figure 8**, there was no publication bias between the included studies when analyzed for the overall thyroid disease in PA [p-values: Egger’s test:0.280 and Begg’s test:0.086]. Funnel plot analysis of Egger’s test and Begg’s test were showed in **Supplementary Figures 6 and 7**.

### 3.7 TSA analysis result

The results of the TSA analysis are shown in **Figure 2D**. This analysis tests the stability of the statistical results by calculating the amount of required information size (RIS) and the bounds and null lines for hypothesis testing. The cumulative z-curve shown in the figure crosses the traditional bounds and the TSA bounds, and although the cumulative amount of information did not reach the desired value, no more tests were needed, proving that the results of this study are trustworthy.

## 4 Discussion

This systematic review and meta-analysis provides the first comprehensive assessment of the relationship between PA and various thyroid diseases. The overall prevalence of thyroid diseases was higher in PA patients than in controls (OR: 1.33, 95% CI: 1.03-1.71, p=0.03). However, subgroup analyses for specific thyroid diseases revealed that this difference was predominantly attributed to a significantly higher prevalence of thyroid nodules in the PA group (OR: 1.85, 95% CI: 1.23-2.80, p=0.003). No statistically significant differences were found between PA and other specific thyroid conditions, including hyperthyroidism, hypothyroidism, thyroiditis, or thyroid cancer.

PA is a common form of secondary hypertension that can lead to severe cardiovascular and metabolic complications if not properly treated (28). Previous studies have found significant negative effects of PA on the cardiovascular and renal systems, including an increased incidence of metabolic syndrome, osteoporosis, and psychiatric disorders (29, 30). A recent study has revealed a significantly elevated prevalence of thyroid nodules among patients with metabolic syndrome, confirming an association between insulin resistance and the formation of thyroid nodules (31). It is plausible that the link we observed is not solely causal but also reflects this shared metabolic dysfunction. Hyperinsulinemia and insulin-like growth factor 1 (IGF-1) are potent mitogens that may stimulate the growth of thyroid cells. Mineralocorticoid receptors (MR) are expressed not only in the kidneys but also in extra-adrenal tissues, including the thyroid gland. Chronic exposure to elevated aldosterone levels may lead to the activation of MR in thyroid follicular cells. This activation can promote pro-fibrotic and pro-inflammatory pathways, which are known to contribute to cell proliferation and nodule formation (32, 33). Additionally, several studies have reported adverse effects of PA on the function and structure of the thyroid gland (34, 35). On the other hand, monitoring bias may be a potentially reasonable non-biological explanation. PA patients are typically managed by endocrinologists, who may be more inclined to perform neck physical examinations and request thyroid ultrasounds. It is worth noting that some of the included studies used systematic thyroid ultrasounds for both PA patients and the control group, which may mitigate the impact of monitoring bias to some extent. However, further studies with larger sample sizes and more homogeneous patient populations are needed to confirm these findings.

The clinical implications of our findings are specific. Given the significantly increased risk of thyroid nodules, clinicians caring for patients with PA should be aware of this comorbidity. This does not, however, justify broad screening for all thyroid diseases. Our data do not support routine thyroid function tests in asymptomatic PA patients, as we found no evidence of increased dysfunction. Similarly, there is no evidence from our study to recommend changes to cancer surveillance practices. A pragmatic approach might involve considering a targeted thyroid ultrasound examination in PA patients, primarily to evaluate for the presence of nodules, particularly if there are other clinical indications (e.g., palpable neck mass, symptoms of compression, or a personal history of head and neck irradiation).

Our study has several strengths. First, we conducted a comprehensive and systematic search of relevant literature to identify all studies that reported the incidence of thyroid diseases in PA patients. This allowed us to include a larger sample size and provide more reliable estimates of the association between PA and thyroid diseases. Second, we used rigorous methods for literature quality assessment, data extraction, and statistical analysis to minimize bias and ensure the validity of our findings. Third, we performed subgroup analyses and sensitivity analyses to explore the heterogeneity of the included studies and assess the robustness of our results.

However, our study also has some limitations. First, the number of included studies was relatively small, which may limit the power of our meta-analysis to detect statistically significant differences in the prevalence of specific thyroid diseases. Second, the included studies were heterogeneous in terms of patient populations, study designs, and outcome measures, which may affect the generalizability of our findings. Third, we were unable to assess the potential confounding effects of other variables, such as age, gender, and coexisting medical conditions, on the association between PA and thyroid diseases due to the limited information available in the included studies. Fourth, existing literature lacks detailed characterization of thyroid nodules. Most studies rely solely on ultrasound without histological confirmation, and data on nodule diameter are extremely limited. Consequently, we cannot draw definitive conclusions about the malignant potential or size distribution of thyroid nodules in PA patients.

## 5 Conclusion

Our study provides evidence for a significant association between PA and thyroid nodules. While an elevated overall risk of thyroid disease was observed, this association was predominantly driven by a markedly higher prevalence of thyroid nodules in PA patients. We did not find statistically significant evidence linking PA to an increased risk of other specific thyroid conditions in the present analysis. These findings highlight the importance of targeted screening for thyroid nodules in patients with PA. Further studies with larger sample sizes and more homogeneous patient populations are needed to confirm the robust link between PA and thyroid nodules and to explore the potential mechanisms underlying this specific association.

## Data Availability

The original contributions presented in the study are included in the article/**Supplementary Material**. Further inquiries can be directed to the corresponding author.
