# Supplementary material for: Prevalence and risk of thyroid disease among adult primary aldosteronism patients: a systematic review, meta-analysis, and trial sequential analysis
Source: Front Endocrinol (Lausanne). 2025 Oct 17;16:1614789. doi: 10.3389/fendo.2025.1614789 (PMC12575143; doi:10.3389/fendo.2025.1614789)
Supplement: Supplementary file 1 [file DataSheet1.docx]

**Search Strategies**

**Database :PubMed**

**Search time :December 01, 2024**

**Strategy :**

1. "Hyperaldosteronism"[Mesh]
2. ((((((((Aldosteronism[Title/Abstract]) OR (Conn Syndrome[Title/Abstract])) OR (Syndrome, Conn[Title/Abstract])) OR (Conn's Syndrome[Title/Abstract])) OR (Conns Syndrome[Title/Abstract])) OR (Syndrome, Conn's[Title/Abstract])) OR (Primary Hyperaldosteronism[Title/Abstract])) OR (Hyperaldosteronism, Primary[Title/Abstract])) OR (Primary aldosteronism[Title/Abstract])
3. #1 OR #2
4. "Thyroid Diseases"[Mesh]
5. (((((Diseases, Thyroid[Title/Abstract]) OR (Disease, Thyroid[Title/Abstract])) OR (thyroid function[Title/Abstract])) OR (Hypothyroidism[Title/Abstract])) OR (Hyperthyroidism[Title/Abstract])) OR (Thyroid Cancer[Title/Abstract])
6. #4 OR #5
7. #3 AND #6

**Database :EMBASE**

**Search time :December 01, 2024**

**Strategy :**

1. 'primary hyperaldosteronism'/exp
2. 'primary aldosteronism':ab,ti OR 'aldosteronism primary':ab,ti OR 'aldosteronism, primary':ab,ti OR 'hyperaldosteronism, primary':ab,ti OR 'idiopathic aldosteronism':ab,ti OR 'primary hyperaldosteronismus':ab,ti OR 'primary hyperaldosteronism':ab,ti OR 'conn syndrome':ab,ti
3. #1 OR #2
4. 'thyroid disease'/exp
5. 'thyroid disease':ab,ti OR 'thyroid diseases':ab,ti OR 'thyroid disorder':ab,ti OR 'thyroid dysfunction':ab,ti OR hypothyroidism:ab,ti OR hyperthyroidism:ab,ti OR 'thyroid cancer':ab,ti
6. #4 OR #5
7. #3 AND #6

**Database :Cochrane**

**Search time :December 01, 2024**

**Strategy :**

1. [Hyperaldosteronism] explode all trees
2. (Hyperaldosteronism):ti,ab,kw OR (Aldosteronism):ti,ab,kw OR (Hyperaldosteronism, Primary):ti,ab,kw OR (Syndrome, Conn):ti,ab,kw OR (Conn Syndrome):ti,ab,kw
3. (Syndrome, Conn's):ti,ab,kw OR (Conn's Syndrome):ti,ab,kw OR (Primary Hyperaldosteronism):ti,ab,kw OR (Conns Syndrome):ti,ab,kw
4. #1 OR #2 OR #3
5. [Thyroid Diseases] explode all trees
6. (thyroid disease):ti,ab,kw OR (thyroid dysfunction):ti,ab,kw OR (Hypothyroidism):ti,ab,kw OR (Hyperthyroidism):ti,ab,kw OR (Thyroid Cancer):ti,ab,kw
7. #5 OR #6
8. #4 AND #7

**Database :Web of Science**

**Search time :December 01, 2024**

**Strategy :**

1. primary aldosteronism (Topic) OR Aldosteronism (Topic) OR Conn Syndrome (Topic) OR Syndrome, Conn (Topic) OR Conn's Syndrome (Topic) OR Conns Syndrome (Topic) OR Syndrome, Conn's (Topic) OR Primary Hyperaldosteronism (Topic) OR Hyperaldosteronism, Primary (Topic) OR Primary aldosteronism (Topic)
2. thyroid disease (Topic) OR thyroid disorder (Topic) OR thyroid dysfunction (Topic) OR Hypothyroidism (Topic) OR Hyperthyroidism (Topic) OR Thyroid Cancer (Topic) OR Thyroid Neoplasm (Topic)
3. #1 AND #2


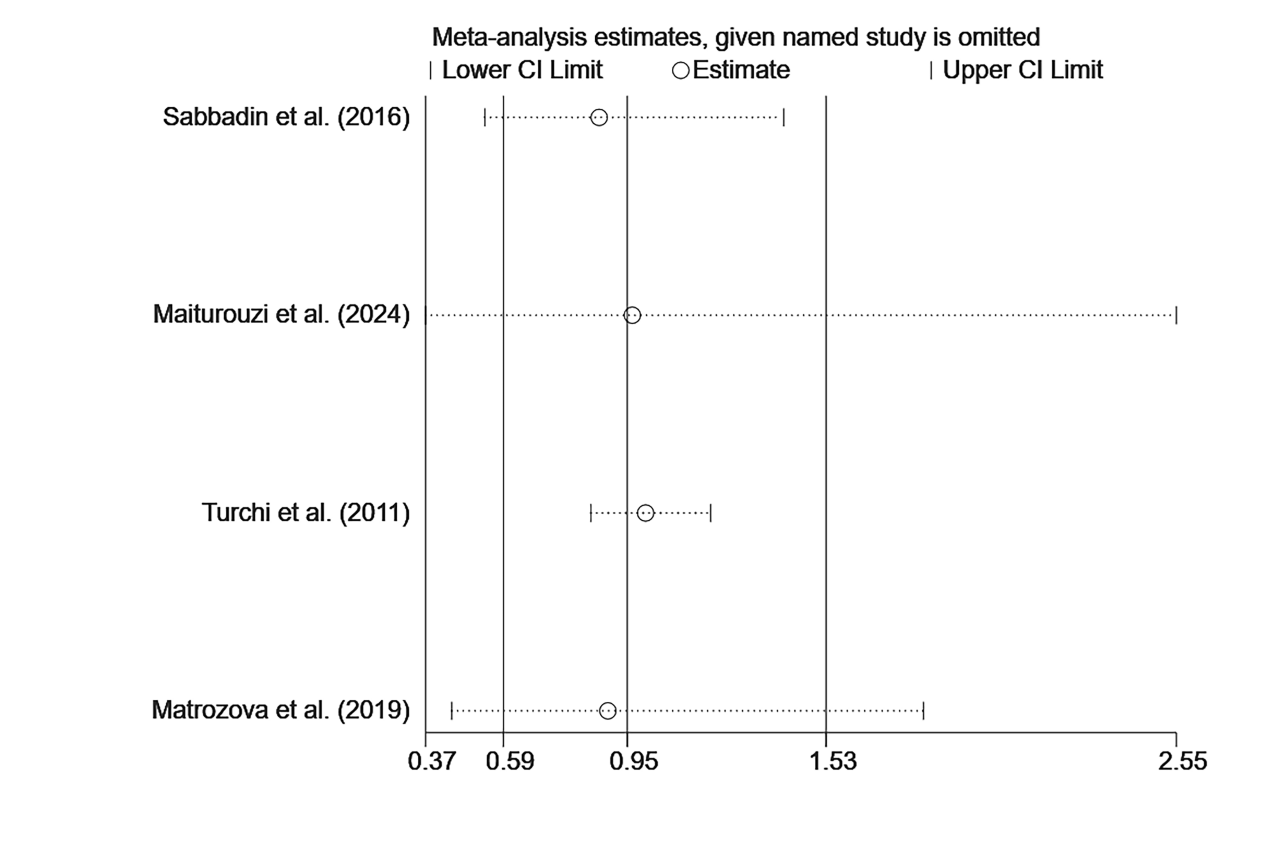


S1


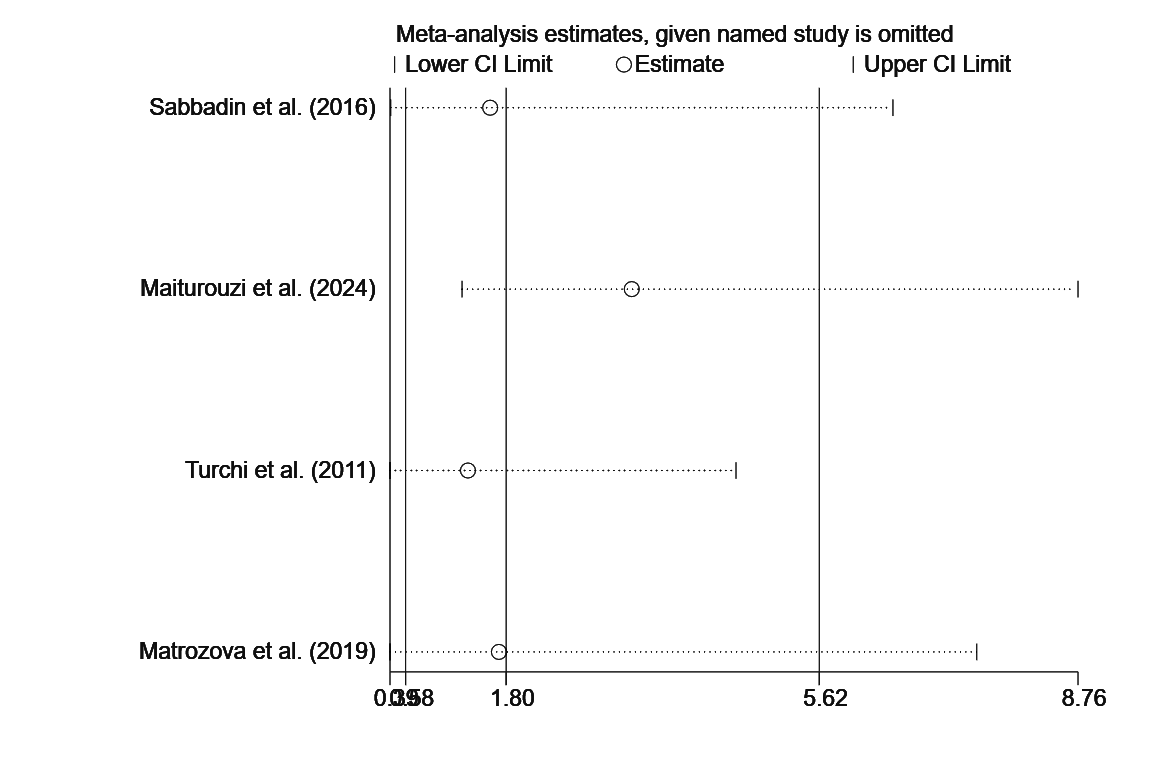


S2


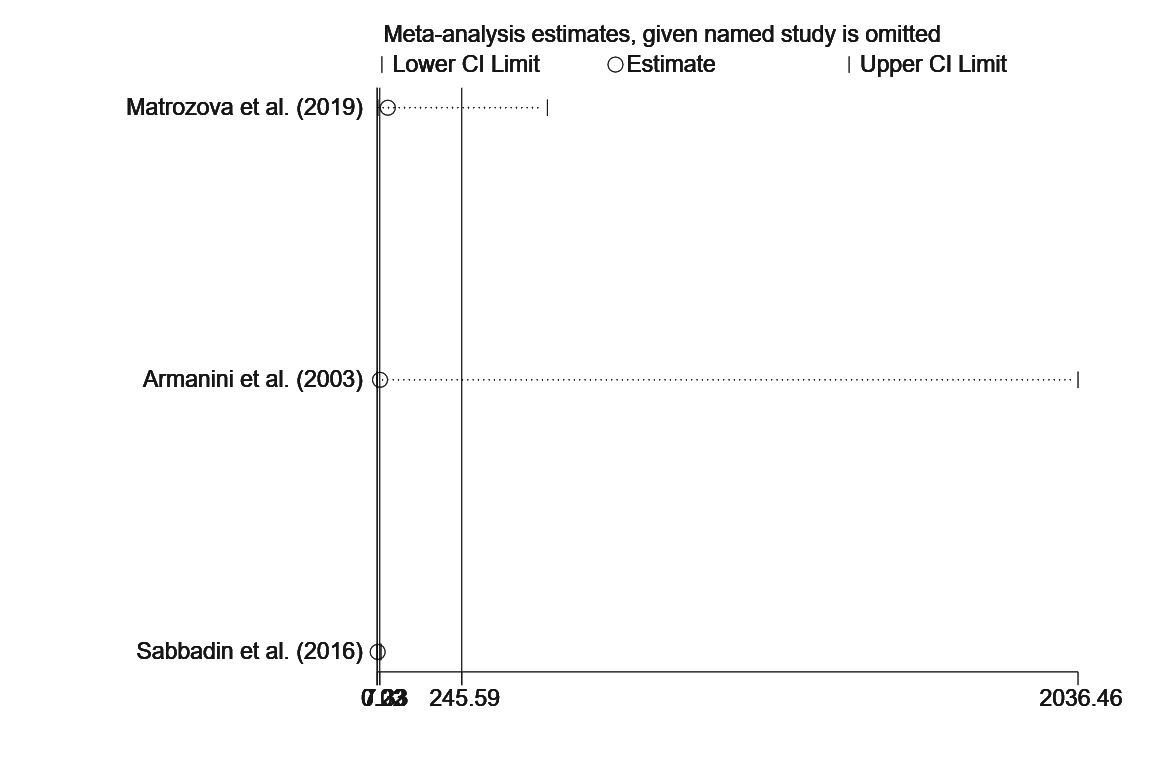


S3


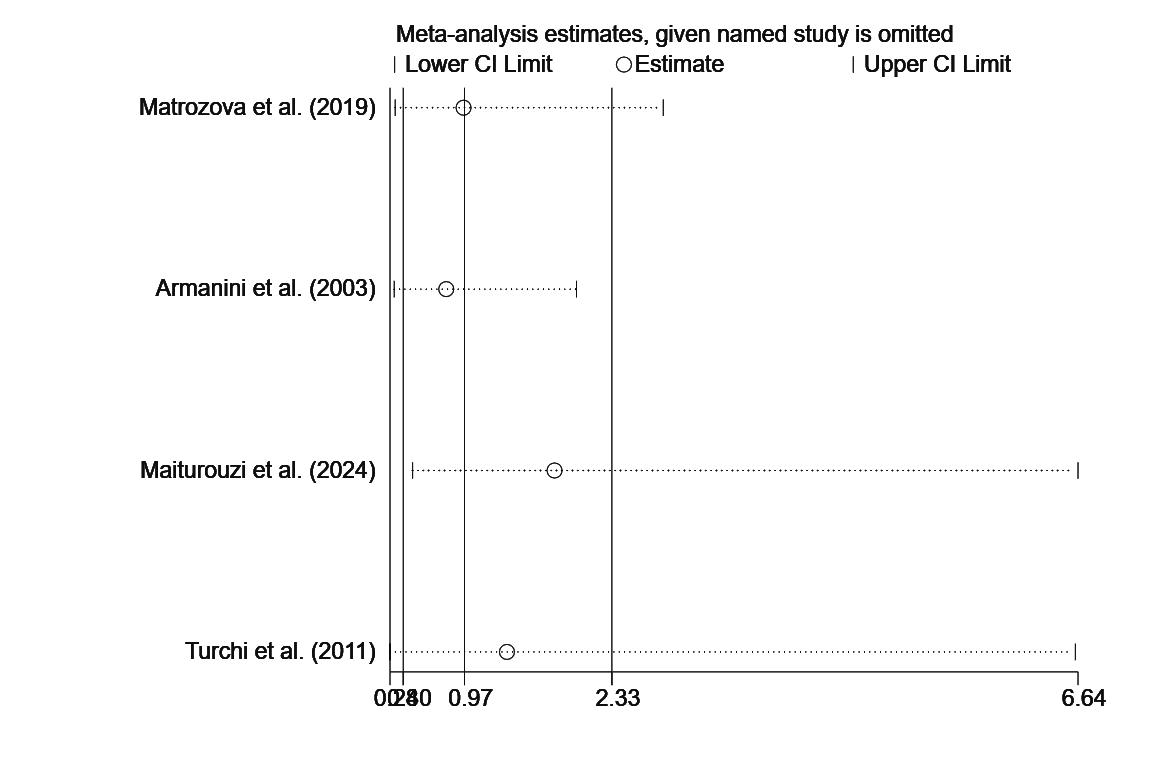


S4


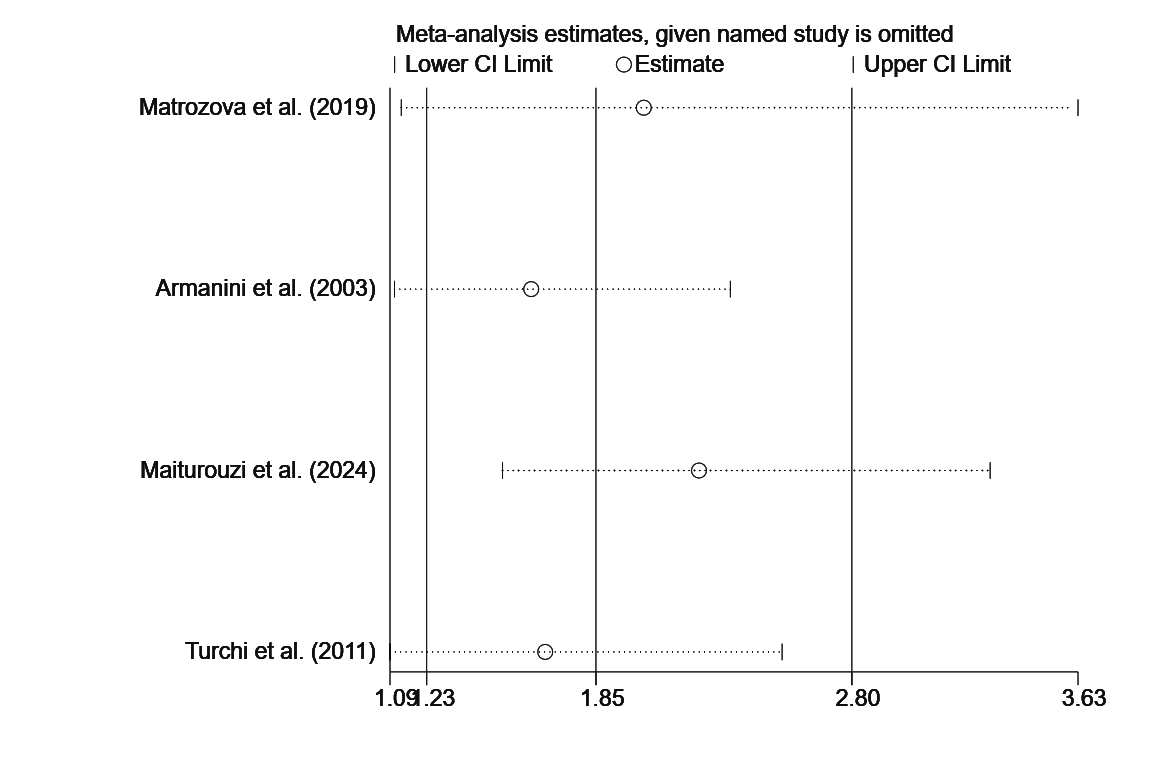


S5


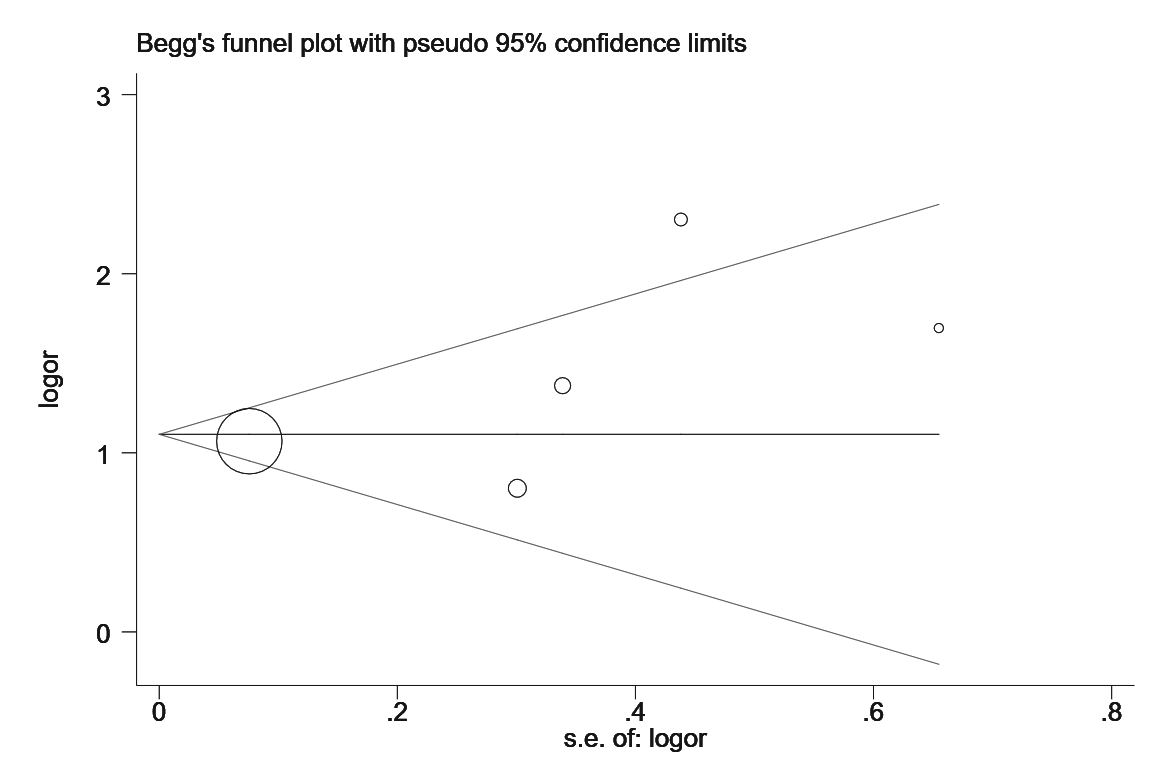


S6


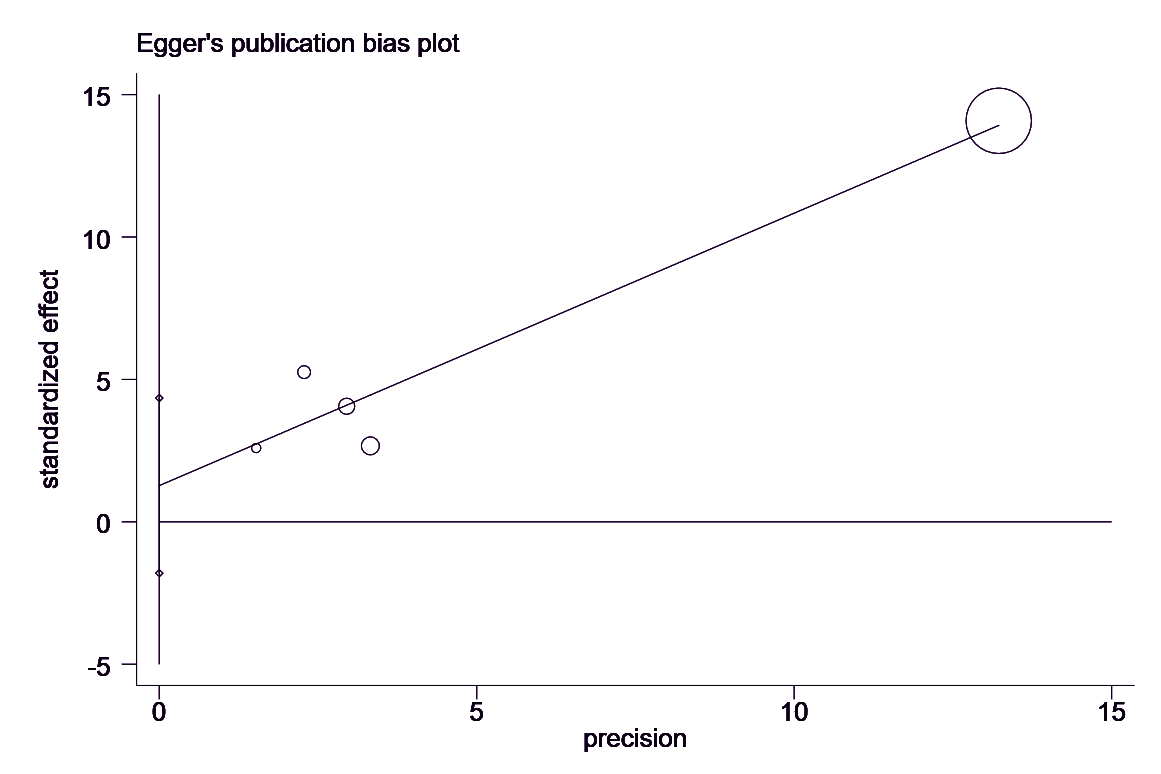


S7


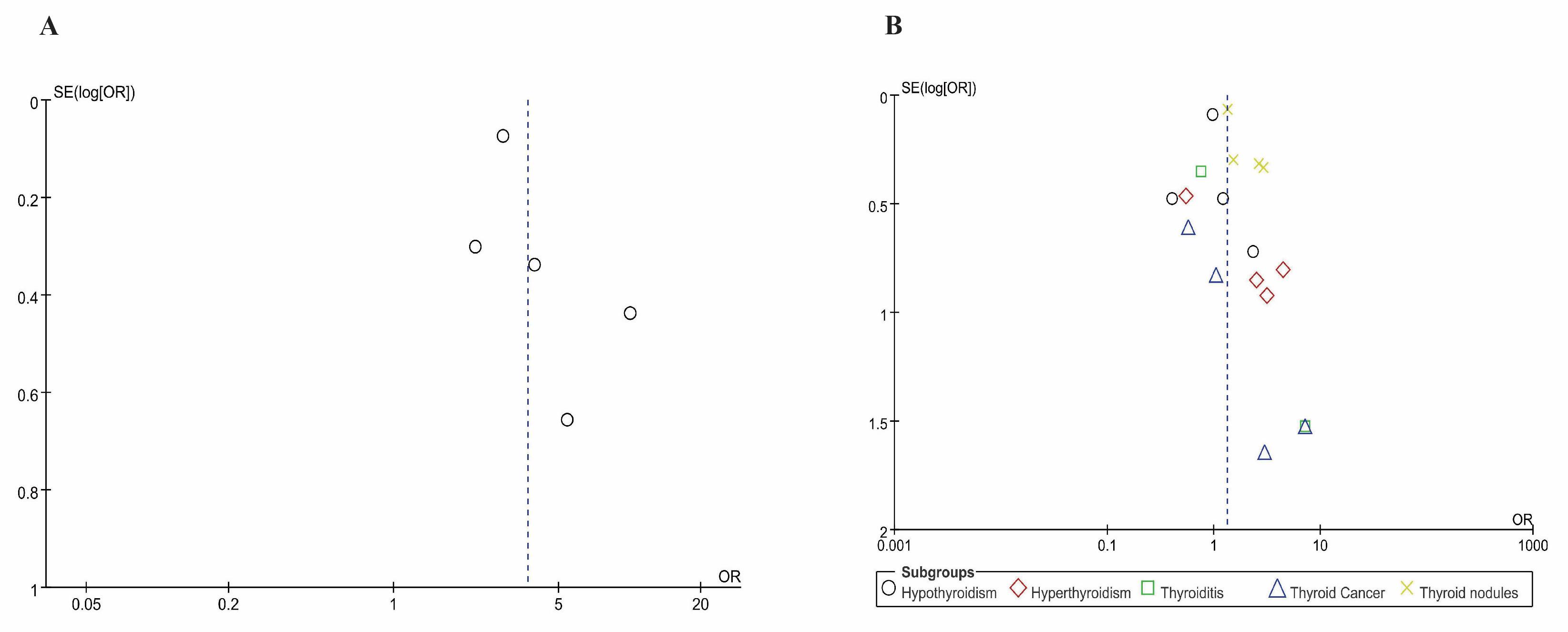


S8
